# Supplementary material for: The interactome of intact mitochondria by cross-linking mass spectrometry provides evidence for coexisting respiratory supercomplexes
Source: Mol Cell Proteomics. 2017 Dec 8;17(2):216–32. doi: 10.1074/mcp.RA117.000470 (PMC5795388; doi:10.1074/mcp.RA117.000470)
Supplement: Supplemental Data [file supp_RA117.000470_133922_0_supp_23978_fzffwf.zip › spectra_annotation/mito_DR_spectra_annotation/411-1-4-1-10-1.pdf]

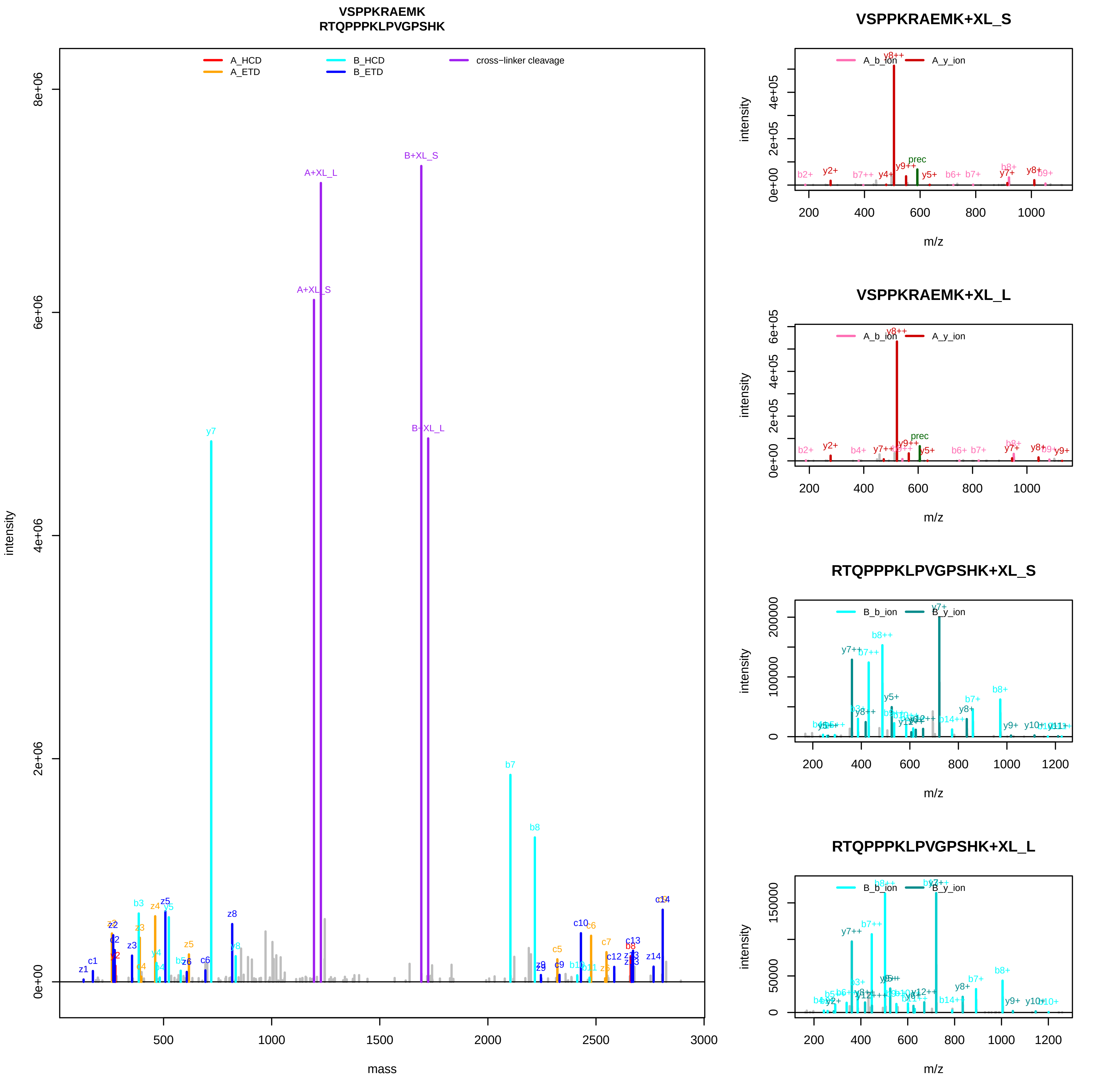
VSPPKRAEMK
RTQPPPKLPVGPSHK
A_HCD
A_ETD
B_HCD
B_ETD
cross−linker cleavage
B+XL_S
A+XL_L
A+XL_S
B+XL_L
y7
b7
b8
c14
c10
c6
c7
z8
z5
b3
z4
c5
intensity
mass
0e+00
2e+06
4e+06
6e+06
8e+06
500
1000
1500
2000
2500
3000
VSPPKRAEMK+XL_S
VSPPKRAEMK+XL_L
RTQPPPKLPVGPSHK+XL_S
RTQPPPKLPVGPSHK+XL_L
A_b_ion
A_y_ion
B_b_ion
B_y_ion
prec
y8++
y9++
y7++
b8++
b7++
b8+
b7+
y8+
y9+
y10+
m/z
intensity
